# Postnatal Consumption of Black Bean Powder Protects against Obesity and Dyslipidemia in Male Adult Rat Offspring from Obese Pregnancies

**DOI:** 10.3390/nu16071029

**Published:** 2024-04-01

**Authors:** Divya Choudhary, Gabriella A. Andreani, Saleh Mahmood, Xiaozhong Wen, Mulchand S. Patel, Todd C. Rideout

**Affiliations:** 1 Department of Exercise and Nutrition Sciences, School of Public Health and Health Professions, State University of New York at Buffalo, Buffalo, NY 14214, USA; divyacho@buffalo.edu (D.C.); gandrean@buffalo.edu (G.A.A.); smahmood@buffalo.edu (S.M.); 2 Department of Pediatrics, Division of Behavioral Medicine, Jacobs School of Medicine and Biomedical Sciences, State University of New York at Buffalo, Buffalo, NY 14214, USA; xiaozhon@buffalo.edu; 3 Department of Biochemistry, Jacobs School of Medicine and Biomedical Sciences, University at Buffalo, Buffalo, NY 14214, USA; mspatel@buffalo.edu

**Keywords:** maternal obesity, offspring developmental programming, dietary pulses, nutritional intervention, dyslipidemia

## Abstract

The adverse influence of maternal obesity on offspring metabolic health throughout the life-course is a significant public health challenge with few effective interventions. We examined if black bean powder (BBP) supplementation to a high-calorie maternal pregnancy diet or a postnatal offspring diet could offer protection against the metabolic programming of metabolic disease risk in adult offspring. Female Sprague Dawley rats were randomly assigned to one of three diets (n = 10/group) for a 3-week pre-pregnancy period and throughout gestation and lactation: (i) a low-caloric control diet (CON); (ii) a high-caloric obesity-inducing diet (HC); or (iii) the HC diet with 20% black bean powder (HC-BBP). At weaning [postnatal day (PND) 21], one male pup from each dam was weaned onto the CON diet throughout the postnatal period until adulthood (PND120). In addition, a second male from the HC group only was weaned onto the CON diet supplemented with BBP (CON-BBP). Thus, based on the maternal diet exposure and offspring postnatal diet, four experimental adult offspring groups were compared: CON/CON, HC/CON, HC-BPP/CON, and HC/CON-BBP. On PND120, blood was collected for biochemical analysis (e.g., lipids, glycemic control endpoints, etc.), and livers were excised for lipid analysis (triglycerides [TG] and cholesterol) and the mRNA/protein expression of lipid-regulatory targets. Compared with the CON/CON group, adult offspring from the HC/CON group exhibited a higher (*p* < 0.05) body weight (BW) (682.88 ± 10.67 vs. 628.02 ± 16.61 g) and hepatic TG (29.55 ± 1.31 vs. 22.86 ± 1.85 mmol/g). Although maternal BBP supplementation (HC-BBP/CON) had little influence on metabolic outcomes, the consumption of BBP in the postnatal period (HC/CON-BBP) lowered hepatic TG and cholesterol compared with the other treatment groups. Reduced hepatic TG in the HC/CON-BBP was likely associated with lower postnatal BW gain (vs. HC/CON), lower mRNA and protein expression of hepatic Fasn (vs. HC/CON), and lower serum leptin concentration (vs. CON/CON and HC groups). Our results suggest that the postnatal consumption of a black-bean-powder-supplemented diet may protect male rat offspring against the programming of obesity and dyslipidemia associated with maternal obesity. Future work should investigate the bioactive fraction of BBP responsible for the observed effect.

## 1. Introduction

Nearly 40% of women of childbearing age in the U.S. have obesity, making maternal obesity a significant public health concern for both mothers and their offspring [1,2]. By programming permanent hypothalamic fetal adaptations (e.g., leptin signaling), maternal obesity contributes to the intrauterine transmission of obesity by altering appetite regulation in offspring [3]. Further, exposure to a maternal obesogenic environment has been shown to induce fetal liver dysfunction (e.g., increased *de novo* lipogenesis, reduced oxidative capacity) and thereby predispose offspring to metabolic-associated fatty liver disease (MASLD) [4,5,6].

Maternal obesity and/or overnutrition can program early-life metabolic dysfunction during in utero fetal development [7,8] and in the early postnatal period through altered maternal milk composition [9,10]. Although the underlying mechanisms are broad, nutritional status is highly influential in programming obesity and is thus a key target for preventative interventions. Nutritional strategies to prevent poor metabolic health outcomes in offspring from obese pregnancies can theoretically be implemented at different life-stages in both mothers and offspring [11]. For instance, in utero fetal development may be protected through improvements in maternal dietary intake throughout gestation [12]. Moreover, pre-pregnancy nutrition interventions in women of childbearing age may be even more advantageous in protecting the health of future offspring [13,14], particularly since roughly half of pregnancies in the U.S. are unplanned [15]. Additionally, nutritional interventions provided to offspring in the postnatal period may be effective in ‘de-programming’ dysfunctional metabolic health in offspring from obese pregnancies [16,17].

Given their high nutrient profile [18] (i.e., protein, fiber, micronutrients, phytochemicals, etc.), the increased consumption of dietary pulses including dried beans, lentils, and peas may be an effective and feasible means to prevent the developmental programming of obesity [19,20]. In non-pregnant populations, pulse consumption has been shown to support a healthy body weight [21,22,23] and influence a broad range of metabolic health outcomes including reduced inflammation [24], improved glycemic control [25], and lower blood lipids [26]. Notably, a recent meta-analysis of 28 cohort studies concluded that dietary pulse consumption is associated with a decreased risk of various chronic conditions, including cardiovascular disease, coronary heart disease, hypertension, and obesity [27]. However, limited research has been conducted on pulse consumption during pregnancy, with little exploration of its potential health-promoting effects during childhood and adolescence [28]. Thus, the objectives of this study were to assess whether black bean powder (BBP) supplementation to mothers (throughout pre-pregnancy, gestation, lactation) or to pre-programmed male offspring would offer protection against obesity and associated metabolic dysfunction (i.e., dyslipidemia and hepatic steatosis) in adult offspring. We chose to only examine male offspring in this study as we have previously reported that female offspring are largely resistant to the metabolic programming influence of maternal obesity in this model with respect to the study outcomes [29,30].

## 2. Materials and Methods

**Figure 1:**
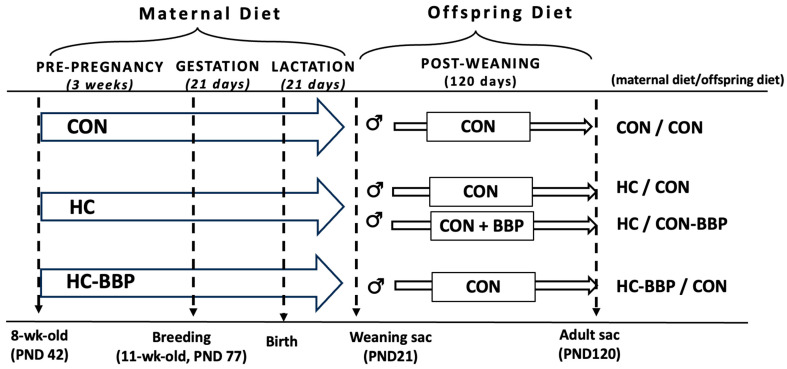
Experimental design. CON, low-calorie control diet; HC, high-calorie obese-inducing diet with casein protein; HC-BBP, high-calorie diet supplemented with black bean powder; PND, postnatal day. Group designations: CON/CON, adult male offspring from CON mothers fed a postnatal CON diet; HC-CON, adult male offspring from HC mothers fed a postnatal CON diet; HC-BBP/CON, adult offspring from HC-BBP mothers fed a postnatal CON diet; HC-CON/BBP, adult male offspring from HC mothers fed a postnatal CON diet supplemented with BBP.

***Animals, diet, and design:*** The animals used in this experiment were cared for in accordance with the guidelines established by the Institutional Animal Care and Use Committee (IACUC). All procedures were reviewed and approved by the Animal Care Committee at the University at Buffalo. The experimental design is provided in Figure 1. 

Thirty-seven-week-old female Sprague Dawley rats (Charles River, obese prone, Crl: OP-CD) were housed in the Laboratory Animal Care Facility at the University at Buffalo and acclimated to the environment for one week prior to the initiation of the experiment. The rats were housed individually in cages with shavings in a temperature-controlled room (20 °C and 50% humidity) with a standard light cycle (12 h light/12 h dark) and given free access to food (chow) and water. Cages and treatments were randomized in the animal room to avoid potential confounding by cage location. Body weights and feed intake were obtained once per week throughout the experiment. Upon the initiation of the experiment, the rats were randomly (random number table) assigned to 1 of 3 diets (n = 10/dietary group, Table 1) for a 3-week pre-pregnancy period and throughout gestation and lactation: (i) a low-caloric control diet (CON; total energy 3.82 kcal/g; % energy from fat 10; protein, 20; and carbohydrate, 70) (Research Diets, D12450K); (ii) a high-caloric obesity-inducing diet (HC; total energy 4.7 kcal/g; % energy from fat, 44; protein, 20; and carbohydrate, 35) (Research Diets, D12451); and (iii) the high-caloric diet supplemented with 20% black bean powder (HC-BBP; total energy 4.56 kcal/g; % energy from fat, 45; protein, 20; and carbohydrate, 34). The ingredients for each diet were mixed on-site every 2 weeks and stored at 4 °C. The HC-BBP diet was adjusted to match the macronutrient and calorie distribution of the HC diet based on external nutrient analyses of the BBP (footnote, Table 1) (Anresco Laboratories, San Francisco, CA, USA). 

Following the pre-pregnancy period, females were timed-mated with CON-fed male breeders for overnight breeding sessions. The next morning, if vaginal plugs or spermatozoa in vaginal lavage were observed, successful mating was confirmed. Body weight and food intake were recorded weekly throughout pregnancy. At birth (postnatal day (PND) 0), the live litter size and weight was recorded. On PND 2, the litters were randomly culled to eight pups per dam (four males and four females where possible) to minimize the variability in pup growth influenced by litter size. Throughout lactation, maternal body weight, food intake, and litter weights were recorded every week. To prevent pup access to the maternal diet, food pellets were placed on raised platforms (accessible only to dams) and cages were changed on a daily basis to minimize fallen scraps of food being available to the pups. 

On PND 21, one male pup from each dam (per diet group) was weaned onto the CON diet throughout the postnatal period until PND 120 (adulthood). In addition, a second male from the HC group only was weaned onto the CON diet supplemented with 40% BBP (CON-BBP, total energy 3.51 kcal/g; % energy from fat 10; protein, 20; and carbohydrate, 70). A 40% supplementation level of BBP was provided based on the higher dose ranges of pulses used in previous non-pregnant rodent model studies [31,32]. A lower BBP dose (20%) was used in the maternal supplemented diet (HC-BBP) to avoid potential interference with nutrient bioavailability [33]. Thus, based on the maternal diet exposure throughout pre-pregnancy, gestation, and lactation (CON, HC, and HC-BBP with 20% BBP) and the postnatal diet consumed (CON or CON-BBP with 40% BBP), 4 experimental adult offspring groups were compared: CON/CON, HC/CON, HC-BPP/CON, and HC/CON-BBP. On PND120, offspring were anesthetized using isoflurane for non-fasting blood collection by decapitation. Additionally, livers were excised, flash frozen in liquid nitrogen, and stored at −80 °C until further processing and analyses.

***Blood biochemistry:*** Serum glucose was measured using colorimetric detection (Invitrogen, Frederick, MD, USA; EIAGLUC) and insulin using ELISA (Millipore, Billerica, MA, USA; EZRMI-13K). Serum cholesterol, including total cholesterol (TC), high-density lipoprotein cholesterol (HDL-C), and LDL/VLDL-C, was measured by enzymatic analysis (Bioassay Systems, Hayward, CA, USA; EHDL-100) and TG were assessed with a commercial enzymatic kit (Zenbio, Durham, NC, USA; STG-1 NC). ELISA was used to quantify serum adiponectin (Crystal Chem, #80570) and leptin (Crystal Chem, #90049).

***Hepatic lipids:*** Hepatic TG were analyzed with a commercial enzymatic kit (ab65336, Abcam, Cambridge, MA, USA) according to the manufacturer’s instructions following TG extraction in aqueous Triton-X buffer (5%). Hepatic cholesterol was extracted and analyzed according to our previously published procedures [34]. Pulverized liver tissue (~0.5 g) was spiked with α-cholestane as an internal standard and saponified in freshly prepared KOH–methanol at 100 °C for 1 h. Petroleum diethyl ether was used to extract the non-saponifiable sterol fraction, which was then dried under N2 gas. A Shimadzu GC-17A gas chromatograph with a flame ionization detector using a SAC-5 capillary column (30 m × 0.25 mm × 0.25 mm, Supelco, Bellefonte, CA, USA) was used to analyze the sterol fractions. 

***mRNA extraction and real time RT-PCR:*** Total RNA was isolated from whole liver tissue (~25 mg) using the RNeasy mini-kit (Qiagen, Germantown, MD, USA, #74104). RNA concentration and integrity was determined with spectrophotometry (260 nm). RNA preparation and real-time RT-PCR were conducted using a one-step QuantiFast SYBR Green RT-PCR kit (Qiagen, #204154) with a Biorad CFX96 Touch real-time PCR system. Gene expression was analyzed using the 2^−ΔΔCt^ method [35]. Validated primer sets (QuantiTect Primer Assays, Qiagen) were used for the following genes: β-actin (*Actb*, *GeneGlobe ID: QT00193473*), fatty acid synthase *(Fasn*, *QT00371210*), acetyl-CoA carboxylase alpha (*Acaca*, *QT00190946*), carnitine palmitoyltransferase 1a (*Cpt1a*, *QT01798825*), 3-hydroxy-3-methylglutaryl-CoA reductase (*Hmgcr*, *QT00182861*), low-density lipoprotein receptor (*Ldlr*, *QT00177744*), sterol regulatory element binding transcription factor 1 (*Srebf1*, *QT02350313*), and sterol regulatory element binding transcription factor 2 (*Srebf2*, *QT00403305*). 

***Western blot:*** Total liver tissue protein was extracted with a commercial kit according to the manufacturer’s instructions (T-Per Tissue protein Extraction, #78510, Thermo Scientific, Waltham, MA, USA). Following homogenization and centrifugation (10,000× *g* for 5 min), supernatants were collected for total protein determination (BioRad protein assay dye, #500-0006). An equal volume of 2X sodium dodecyl sulfate loading buffer was added to each sample and boiled for 5 min for subsequent Western analysis. Total protein extracts were probed with commercial antibodies specific for Fasn (Cell Signaling, Danvers, MA, USA, #3180) and Acaca (Cell Signaling, #3676). Target proteins were normalized to β-actin and quantified using Image lab (version 4.1, Biorad Laboratories, Hercules, CA, USA).

***Statistical Analysis:*** All statistical analyses were performed using SPSS 16 for Mac (SPSS Inc, Chicago, IL, USA). Data were checked for normality using the Shapiro–Wilk test. Based on our previous work [30], we estimated the requirement of a sample size of 8 mothers/group (effect size, 1.49; power, 0.90; *p* < 0.05) to observe a 10% reduction in offspring body weight. Differences between treatments (with an individual rat as the experimental unit) were analyzed with a general linear model ANOVA with an LSD (least significance difference) post hoc test. Repeated measures analysis was used to assess the trajectory of litter growth throughout lactation and postnatal offspring growth. Lab technicians were aware of the group allocations and treatments during the animal experiment but were blinded during sample analyses. Data are presented as means ± SE (10 mothers/group with no exclusions), and outcome differences were assessed at a significance level of *p* ≤ 0.05. 

## 3. Results

**Figure 2:**
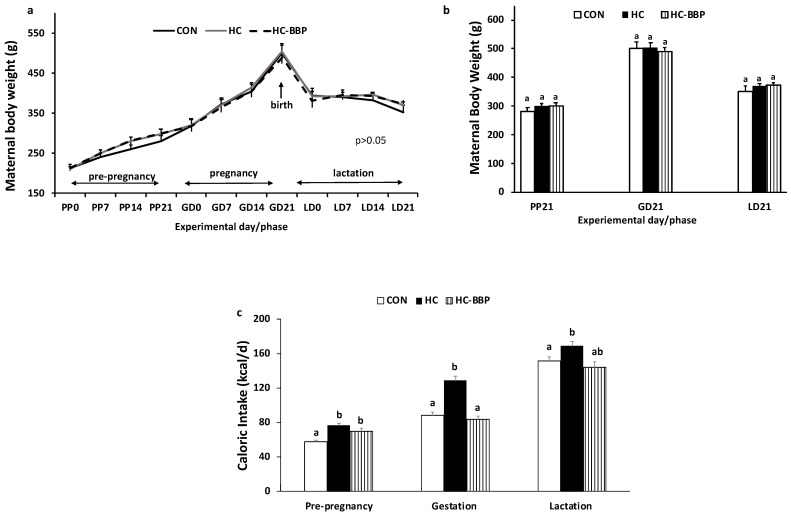
Maternal body weight (g) and caloric intake (kcal/d) throughout pre-pregnancy, gestation, and lactation. Weekly body weight (g) trajectory throughout the experiment (**a**), final body weight (g) at the end of each phase (**b**), and caloric intake (kcal/d) (**c**) throughout pre-pregnancy, gestation, and lactation. PP, pre-pregnancy. GD, gestation day; LD, lactation day; CON, low-calorie control diet; HC, high-calorie obesity-inducing diet with casein protein; HC-BBP, high-calorie diet supplemented with black bean powder. Data are means ± SE; n = 10 mothers per group. Differences between groups were assessed with repeated measures analysis with an LSD (least significance difference) post hoc test; ^ab^ treatment groups that do not share a superscript are significantly different (*p* < 0.05).

***Maternal and litter characteristics:*** Maternal body weight did not differ (*p* > 0.05) between the CON, HC, and HC-BBP groups throughout pre-pregnancy, gestation, and lactation (Figure 2a,b). Compared with CON, HC dams demonstrated increased caloric intake during pre-pregnancy, gestation, and lactation. Maternal BBP supplementation (HC-BBP) normalized caloric intake similar to the CON group in gestation but did not influence caloric intake in pre-pregnancy and lactation relative to the HC group (Figure 2c). 

**Figure 3:**
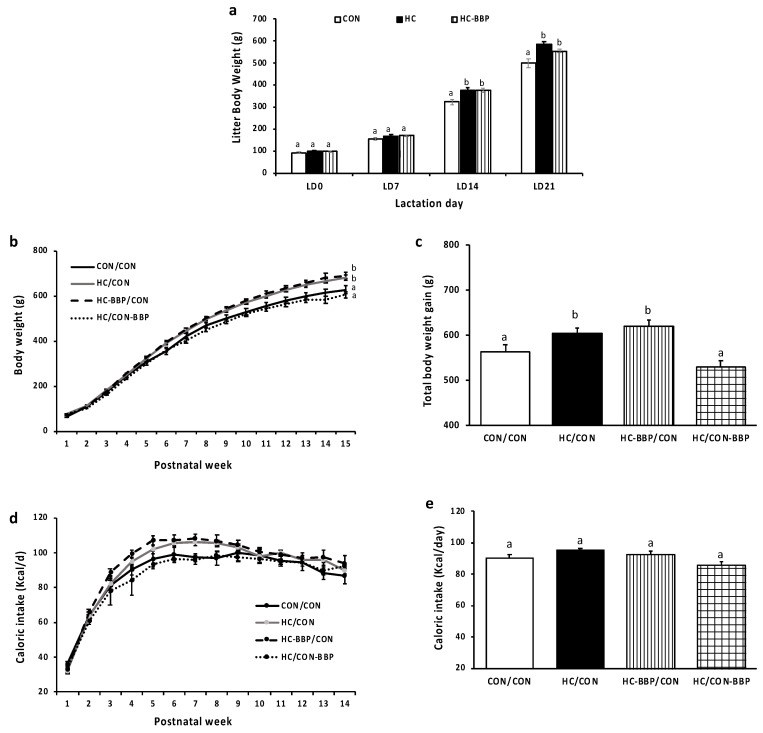
Offspring growth and feed intake. Offspring growth and feed intake. (**a**) Litter weights (g) throughout lactation; (**b**) postnatal body weight gain (g); (**c**) total body weight gain (g); (**d**) trajectory of postnatal caloric intake (kcal/d); (**e**) total postnatal caloric intake (kcal/d). Data are means ± SE; n = 10 mothers per group. Trajectory of body weight and caloric intake was assessed with repeated measures analysis and total body weight and caloric intake was assessed with general linear model ANOVA. An LSD (least significance difference) post hoc test was used to assess differences between groups; ^ab^ treatment groups that do not share a superscript are significantly different (*p* < 0.05).

***Offspring growth and caloric intake:*** Repeated measures analysis demonstrated a higher (*p* < 0.05) trajectory of litter weight gain in HC and HC-BBP dams (vs. CON), driven by increases in LD14 and LD21. No difference (*p* < 0.05) in litter weight was observed between the HC and HC-BBP dams (Figure 3a). The trajectory of postnatal body weight gain was higher (*p* < 0.05) in HC/CON males compared with CON/CON male offspring (Figure 3b). Although maternal BBP supplementation (HC-BBP) did not protect (*p* < 0.05) against the programming of obesity in offspring, BBP supplementation in the postnatal period (HC/CON-BBP) lowered (*p* < 0.05) body weight gain compared with the HC/CON and HC-BBP/CON groups. Total body weight gain showed a similar response between the treatment groups (Figure 3c). Caloric intake in male offspring throughout the postnatal period did not differ (*p* < 0.05) between groups (Figure 3d,e). 

**Figure 4:**
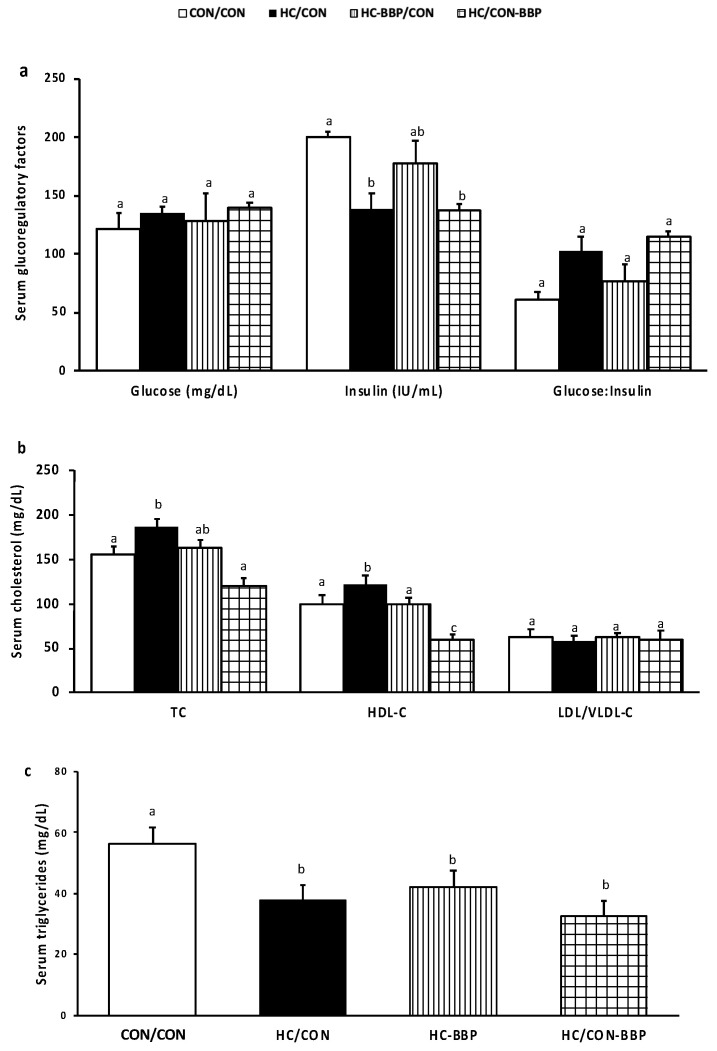
Serum glucoregulatory factors (**a**), cholesterol (**b**), and triglycerides (**c**) in adult male offspring. Outcome parameters were assessed with general linear model ANOVA and an LSD (least significance difference) post hoc test. Data are means ± SE; n = 10 (1 male offspring from each of 10 mothers per group); ^ab^ treatment groups that do not share a superscript are significantly different (*p* < 0.05). CON/CON, adult male offspring from CON mothers fed a postnatal CON diet; HC-CON, adult male offspring from HC mothers fed a postnatal CON diet; HC-BBP/CON, adult offspring from HC-BBP mothers fed a postnatal CON diet; HC-CON/BBP, adult male offspring from HC mothers fed a postnatal CON diet supplemented with BBP.

***Serum biochemical analyses:*** Male offspring from HC/CON and HC/CON-BBP demonstrated lower (*p* < 0.05) insulin concentrations compared with the CON/CON group; however, no treatment effects (*p* > 0.05) were observed for blood glucose or the glucose/insulin ratio (Figure 4a). Adult male offspring from HC/CON dams were dyslipidemic, demonstrating higher (*p* < 0.05) serum total and HDL-C compared with offspring from lean mothers (CON/CON) (Figure 4b). Supplementation of the maternal diet with BBP (HC-BBP/CON) decreased HDL-C in male offspring compared with the HC/CON group. Postnatal BBP consumption (HC/CON-BBP) reduced (*p* < 0.05) serum total-C (vs. HC/CON) and serum HDL-C (vs. the other treatment groups). No difference in LDL/VLDL-C was observed amongst the treatment groups (Figure 4b). Compared with CON/CON, all treatment groups demonstrated lower (*p* < 0.05) serum TG, with no difference observed between the HC/CON, HC-BBP, and HC/CON-BBP groups (Figure 4c). 

**Figure 5:**
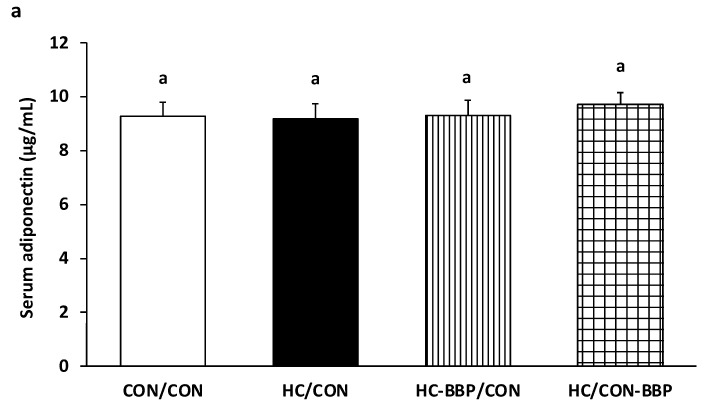

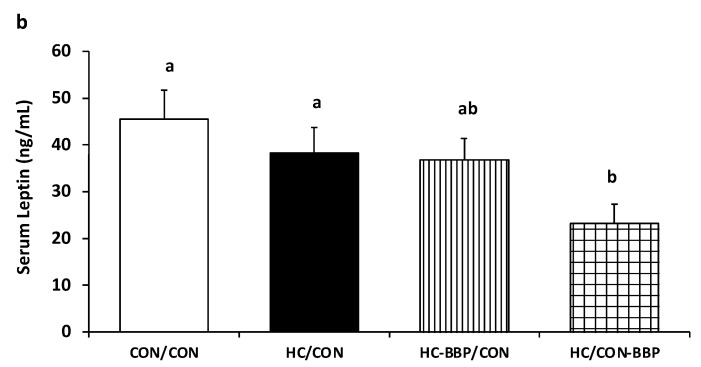
Serum adiponectin (**a**) and leptin (**b**). Outcome parameters were assessed with general linear model ANOVA and an LSD (least significance difference) post hoc test. Data are means ± SE; n = 10 (1 male offspring from each of 10 mothers per group); ^ab^ treatment groups that do not share a superscript are significantly different (*p* < 0.05). CON/CON, adult male offspring from CON mothers fed a postnatal CON diet; HC-CON, adult male offspring from HC mothers fed a postnatal CON diet; HC-BBP/CON, adult offspring from HC-BBP mothers fed a postnatal CON diet; HC-CON/BBP, adult male offspring from HC mothers fed a postnatal CON diet supplemented with BBP.

No difference was observed in serum adiponectin amongst the treatment groups (Figure 5a); however, postnatal BBP supplementation (HC/CON-BBP) reduced (*p* < 0.05) the serum leptin concentration in male offspring compared with the CON/CON and HC/CON groups (Figure 5b). 

**Figure 6:**
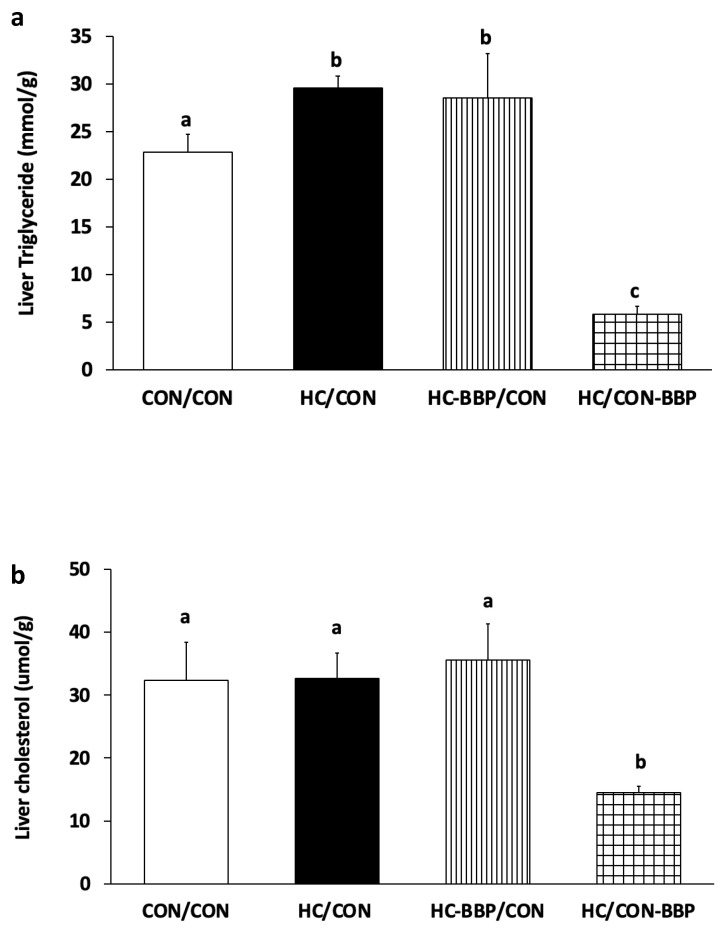
Hepatic triglycerides (mmol/g) (**a**) and cholesterol (μmol/g) (**b**). Outcome parameters were assessed with general linear model ANOVA and an LSD (least significance difference) post hoc test. Data are means ± SE; n = 10 (1 male offspring from each of 10 mothers per group); ^ab^ treatment groups that do not share a superscript are significantly different (*p* < 0.05). CON/CON, adult male offspring from CON mothers fed a postnatal CON diet; HC-CON, adult male offspring from HC mothers fed a postnatal CON diet; HC-BBP/CON, adult offspring from HC-BBP mothers fed a postnatal CON diet; HC-CON/BBP, adult male offspring from HC mothers fed a postnatal CON diet supplemented with BBP.

***Hepatic lipids:*** Male offspring from HC/CON demonstrated increased hepatic TG, but not cholesterol, compared with those from lean mothers (CON/CON). Postnatal BBP supplementation (HC/CON-BBP) reduced (*p* < 0.05) the concentration of both TG and cholesterol compared with the other treatment groups (Figure 6a,b). 

**Figure 7:**
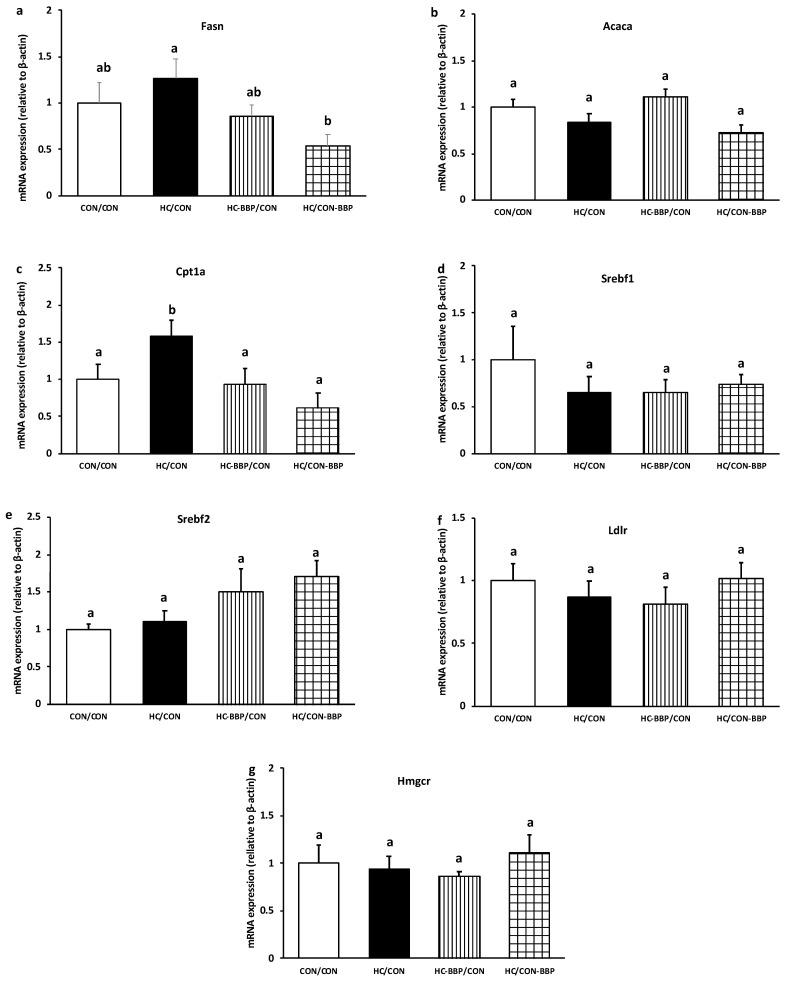
Hepatic mRNA expression of lipid regulatory targets. Hepatic mRNA expression of lipid regulatory targets including (**a**) fatty acid synthase (Fasn); (**b**) acetyl-CoA carboxylase (Acaca); (**c**) carnitine palmitoyltransferase Ia (CPT1a), (**d**) sterol regulatory element-binding transcription factor 1 (SREBP1c), (**e**) sterol regulatory element-binding transcription factor 2 (SREBP2), (**f**) low-density lipoprotein receptor (LDLr), and (**g**) 3-hydroxy-3-methyl-glutaryl-coenzyme A reductase (HMG-CoAr). Outcome parameters were assessed with general linear model ANOVA and an LSD (least significance difference) post hoc test. Data are means ± SE; n = 10 (1 male offspring from each of 10 mothers per group); ^ab^ treatment groups that do not share a superscript are significantly different (*p* < 0.05). CON/CON, adult male offspring from CON mothers fed a postnatal CON diet; HC-CON, adult male offspring from HC mothers fed a postnatal CON diet; HC-BBP/CON, adult offspring from HC-BBP mothers fed a postnatal CON diet; HC-CON/BBP, adult male offspring from HC mothers fed a postnatal CON diet supplemented with BBP.

**Figure 8:**
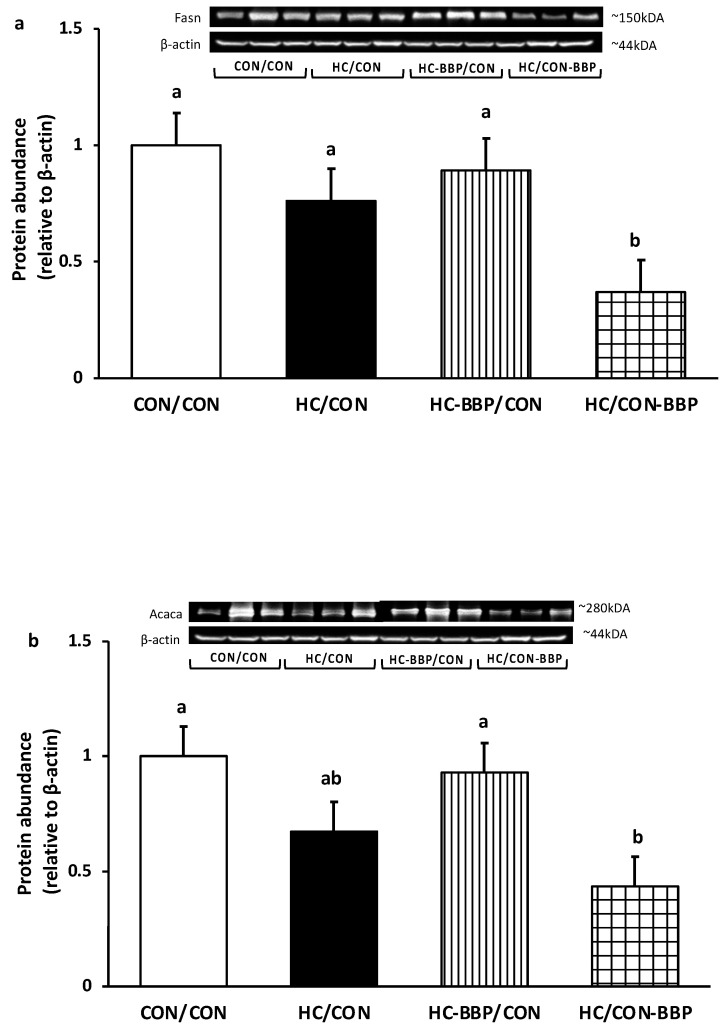
Hepatic protein abundance of (**a**) fatty acid synthase (Fasn) and (**b**) acetyl-CoA carboxylase (Acaca). Data are means ± SE; n = 10 (1 male offspring from each of 10 mothers per group); ^ab^ treatment groups that do not share a superscript are significantly different (*p* < 0.05). CON/CON, adult male offspring from CON mothers fed a postnatal CON diet; HC-CON, adult male offspring from HC mothers fed a postnatal CON diet; HC-BBP/CON, adult offspring from HC-BBP mothers fed a postnatal CON diet; HC-CON/BBP, adult male offspring from HC mothers fed a postnatal CON diet supplemented with BBP.

***Hepatic mRNA and protein expression:*** mRNA expression of Fasn was reduced (0.54-fold of CON/CON, *p* < 0.05) in HC/CON-BBP compared with the HC/CON group (Figure 7a); however, no difference (*p* > 0.05) was observed in Acaca expression between treatment groups (Figure 7b). Adult male offspring from the HC/CON group demonstrated higher (*p* < 0.05) expression of Cpt1a (1.58 of CON/CON) compared with the other treatment groups (Figure 7c). No differences (*p* > 0.05) were noted in the mRNA expression of Srebf1, Srebf2, Ldlr, or Hmgcr amongst the treatment groups (Figure 7d–g). Adult male offspring from HC/CON-BBP demonstrated lower (*p* < 0.05) protein abundance of both Fasn (0.37 of CON/CON vs. all groups) and Acaca (0.43 of CON/CON vs. CON/CON and HC-BBP/CON) (Figure 8a,b).

## 4. Discussion

In the study, we observed that adult male offspring from HC/CON dams had a higher postnatal body weight gain, higher serum total cholesterol, and increased hepatic TG compared with offspring from CON/CON dams. Maternal black bean powder supplementation throughout pre-pregnancy, lactation, and gestation did not protect against the programming of obesity in offspring. However, compared with all other groups, BBP supplementation to a postnatal diet was associated with several positive outcomes including reduced body weight, lower serum total cholesterol, and lower liver cholesterol and TG. 

The prevalence of MASLD has been rapidly increasing, with a current global prevalence of 25% [36,37]. MASLD begins with an excess accumulation of hepatic fat (simple steatosis) [38] and progresses in a sub-set of patients (~20%) to steatohepatitis [39] with an underlying state of heightened inflammation and oxidative stress, with or without fibrosis [40,41]. In addition to the accumulation of liver fat, MASLD is further associated with disturbances in cholesterol metabolism [42,43,44], including increased cholesterol deposition [45] and synthesis [46,47], and the altered expression of cholesterol regulatory genes [48,49]. Although obesity and insulin resistance are closely linked to the pathophysiology of MASLD [50], recent work suggests that there may be a potential programming influence of maternal obesity that predisposes offspring to dysregulated hepatic fat metabolism and MASLD development [5,51,52]. Similarly, we observed that HC/CON offspring had a ~29% increase in liver TG concentration compared with offspring from CON/CON mothers. Given this link, maternal dietary interventions prior to and/or during pregnancy that reduce maternal adiposity may beneficially influence offspring liver function. Accordingly, data from a previous animal model study reported that improving maternal pre-pregnancy nutrition by switching from a high-fat to a standard-fat diet protects offspring from MASLD development [14]. Further, increased maternal fiber intake during pregnancy was associated with lower offspring hepatic fat in early childhood in a recent longitudinal cohort study of 278 mother–child pairs [53]. However, in the current study, the maternal BBP intervention (HC-BBP/CON) did not improve any aspect of metabolic health in offspring. This may be attributed to the limited impact of the intervention on maternal obesity. However, in a similar rat model, we have recently reported that the supplementation of an obesity-inducing maternal diet with yellow pea protein [30] and fiber [29] fractions throughout pregnancy improved the metabolic health of offspring independent of a change in maternal obesity status. 

Although maternal BBP supplementation did not protect against hepatic fat infiltration, postnatal BBP supplementation did result in a reduction in both hepatic TG and cholesterol. This is supported by several previous reports suggesting a potential role of pulses in MASLD development. Recently, Feng et al. (2023) [54] reported that C57/BL6J male mice fed a high-fat diet supplemented with (20%) white kidney bean powder for 12 weeks demonstrated a reduction in hepatic total cholesterol and TG compared with mice that were fed a high-fat diet alone, possibly by normalizing high-fat-diet-induced dysbiosis. Likewise, another previous study in a similar C57/BL6J mouse model reported a protective effect of mung bean protein isolate in reducing liver TG accumulation in male mice fed an atherogenic MASLD-promoting diet [55]. Parallel experiments suggested that the TG-reducing effect of the mung bean protein isolate was not due to the amino acid composition of the isolate per se, but perhaps a bioactive peptide fraction. However, at this time, the specific bioactive component on the BBP that may have contributed the observed protective effect is not known but should be investigated through the examination of the metabolic health effects of individual BBP fractions (i.e., protein, fiber, etc.).

Based on our current data, several contributing factors may underly the hepatic TG and cholesterol-lowering response observed in offspring consuming a postnatal BBP-supplemented diet. First, MASLD is tightly linked with excess adiposity and significant weight loss through diet and exercise interventions, which have proven to be effective in reducing intrahepatic fat content and improving lipotoxicity [56]. Although offspring from HC/CON dams demonstrated increased body weight as adults (vs. CON/CON), postnatal BBP protected against this response, normalizing body weight to near that of the CON/CON group. This response is striking given that the postnatal diets were formulated to be energy balanced. Whether the postnatal BBP intervention ‘deprogramed’ or merely masked the obesogenic influence of maternal obesity is not clear at this time. Regardless, previous work in both animals [57] and humans [22] has demonstrated anti-obesity responses following pulse consumption. 

Second, the hepatic TG-lowering response may be related to a reduction in hepatic lipogenesis, as evidenced by a lower mRNA expression of Fasn and the lower protein abundance of both Fasn and Acaca. A previous study reported that mung bean protein isolate suppressed lipogenic gene expression (Fasn, Srebf1, and scd1) and prevented hepatic TG accumulation in male mice [55]. As previous work suggests that an enhanced *de novo* lipogenic response in MASLD patients contributes to steatosis [58], strategies that target hepatic lipogenesis have received attention as potential therapeutic interventions [59].

Finally, although leptin is most often regarded as an adipose-derived satiety signal, it also exerts direct anti-steatotic effects in the liver by preventing hepatic lipogenesis, stimulating β-oxidation, and promoting VLDL secretion [60]. However, obesity is often associated with defective leptin signaling and a resistance to its typical anti-steatotic effect, leading to the development of hepatic steatosis [61]. This may be a contributing factor to the enhanced hepatic TG observed in HC/CON vs. CON/CON offspring. Alternatively, male offspring consuming the BBP-supplemented diet (HC/CON-BBP) demonstrated a reduction in serum leptin concentration. Similarly, Tan et al. (2021) reported a 43% reduction in serum leptin in male mice fed a high-fat diet (HF; 46% energy from fat) supplemented with (20%) cooked black turtle beans that was also associated with a reduction in hepatic TG [62]. Together, it seems plausible that an as-yet-unknown bioactive component of beans may result in an improvement in leptin sensitivity with direct protective implications for liver lipid signaling and steatosis. 

## 5. Conclusions

In conclusion, our findings suggest that postnatal (but not maternal) BBP supplementation in pre-programmed male offspring is associated with favorable metabolic outcomes, including reduced body weight and improved serum and hepatic lipid profile that may be driven by multiple mechanisms including lower adiposity, reduced lipogenesis, and improved leptin sensitivity. Although these results suggest a potential role of BBP in improving the long-term metabolic health of offspring from obese mothers, our study findings are limited in that we did not examine potential long-term impacts on female offspring or maternal postpartum health. 

## Figures and Tables

**Table 1:** Formulation of experimental diets (% composition; *w*/*w*).

Ingredient	Experimental Diets ^1^			
	CON	HC	HC-BBP	CON-BBP
Casein	18.96	23.31	18.00	7.00
Corn Starch	52.13	8.33	7.00	22.88
Maltodextrin	14.22	11.65	10.00	14.22
Sucrose	0.38	20.60	10.00	0.38
Cellulose	4.74	5.83	5.83	6.74
**Black bean powder**	**0.00**	**0.00**	**20.00**	**40.00**
Soybean Oil	2.37	2.91	2.91	2.00
Lard	1.90	20.69	19.85	1.50
Cholesterol	0.00	0.15	0.15	0.00
Mineral Mix	4.74	5.83	5.83	4.74
Vitamin Mix	0.09	0.12	0.12	0.09
L-Cystine	0.28	0.35	0.35	0.28
Choline Bitartrate	0.19	0.23	0.23	0.19
				
**Energy contribution**				
Total energy (Kcal/g)	3.82	4.69	4.56	3.51
% energy from fat	10.04	45.26	45.36	10.18
% energy from protein	20.13	20.16	20.59	20.02
% energy from Carbohydrate	69.82	34.59	34.06	69.82
				
**Fiber Content (%)**				
Total fiber	4.74	5.83	4.25	6.74

^1^ CON, low-calorie control diet was based on D12450 formulation (Research Diets); HC, high-calorie obesity-inducing diet was based on D12451 (Research Diets); HC-BBP diet supplemented with black bean powder was fed to mothers throughout pre-pregnancy, gestation, and lactation; CON-BBP, CON diet supplemented with black bean powder (adjusted to achieve the desired level of fat, protein, and carbohydrate) was fed to male offspring throughout postnatal period. Nutrient composition of the BBP: moisture, 9.82 g; ash, 4.08 g; fat, 1.16 g; protein, 25.66 g; total carbohydrate, 59.28 g; total dietary fiber, 21.25 g; calories, 3.5 kcal/g).

## Data Availability

The data presented in this study are available on request from the corresponding author. The data are not publicly available due to privacy.
